# Network Pharmacology Analysis of Glycyrrhetinic Acid in Metabolic Dysfunction-Associated Steatotic Liver Disease

**DOI:** 10.3390/metabo16050301

**Published:** 2026-04-29

**Authors:** Osmar Antonio Jaramillo-Morales, Refugio Cruz-Trujillo, Citlaly Natali De la Torre-Sosa, Josselin Carolina Corzo-Gómez, Dulce Concepción Domínguez-Cruz, Raúl Edgardo Cruz-Cadena, Nereida Violeta Vega-Cabrera, Josue Vidal Espinosa-Juárez

**Affiliations:** 1 Departamento de Enfermería y Obstetricia, División de Ciencias de la Vida, Campus Irapuato-Salamanca, Universidad de Guanajuato, Irapuato 36500, Guanajuato, Mexico; oa.jaramillo@ugto.mx (O.A.J.-M.); nv.vega@ugto.mx (N.V.V.-C.); 2 Escuela de Ciencias Químicas, Universidad Autónoma de Chiapas, Ocozocoautla de Espinosa 29140, Chiapas, Mexico; refugio.cruz@unach.mx (R.C.-T.); citlaly.torre@unach.mx (C.N.D.l.T.-S.); josselin.corzo@unach.mx (J.C.C.-G.); dulce.dominguez@unach.mx (D.C.D.-C.); cruz.raul@unach.mx (R.E.C.-C.); 3 Grupo de Investigación en Salud y Metabolitos Especializados con Potencial Farmacológico, Departamento de Químicos Farmacobiólogos, Universidad Pablo Guardado Chávez (UPGCH), Libramiento Norte Oriente No. 3450, Tuxtla Gutiérrez 29040, Chiapas, Mexico; 4 Departamento de Químicos Farmacobiólogos, Universidad Pablo Guardado Chávez (UPGCH), Libramiento Norte Oriente No. 3450, Tuxtla Gutiérrez 29040, Chiapas, Mexico

**Keywords:** glycyrrhetinic acid, MASLD, network pharmacology, molecular docking, inflammation, metabolic regulation

## Abstract

**Background:** Metabolic dysfunction-associated steatotic liver disease (MASLD) is a multifactorial disorder driven by tightly interconnected metabolic, inflammatory, and lipid dysregulation pathways. Glycyrrhetinic acid, a pentacyclic triterpenoid derived from *Glycyrrhiza* species, has demonstrated anti-inflammatory and hepatoprotective activities in previous experimental studies. **Objectives**: This study aimed to systematically investigate the potential molecular targets and signaling pathways of glycyrrhetinic acid in MASLD using an integrated network pharmacology and molecular docking strategy. **Methods**: Predicted protein targets of glycyrrhetinic acid and MASLD-associated genes were collected from public databases. A protein–protein interaction (PPI) network was constructed, and hub genes were identified using the maximal clique centrality algorithm in Cytoscape. Functional annotation was performed through Gene Ontology and KEGG pathway enrichment analyses. Molecular docking simulations were subsequently conducted to assess the binding affinity of glycyrrhetinic acid with biologically prioritized targets derived from the network analysis. **Results**: Intersection analysis identified 26 shared targets between glycyrrhetinic acid and MASLD. PPI network analysis highlighted IL6, TNFα, AKT1, and PPARγ as central hub genes. Functional enrichment indicated that these targets were mainly involved in NF-κB, TNFα, and PI3K–Akt signaling pathways. Molecular docking results revealed favorable predicted binding affinities, with glycyrrhetinic acid exhibiting the strongest binding toward PPARγ among the evaluated targets. **Conclusions**: This integrative in silico analysis suggests that glycyrrhetinic acid may interact with multiple MASLD-related targets involved in inflammatory and metabolic regulation. These findings provide a computational framework for target prioritization and support further experimental investigations to elucidate the pharmacological relevance of glycyrrhetinic acid in MASLD.

## 1. Introduction

Metabolic dysfunction-associated steatotic liver disease (MASLD) has emerged as the most prevalent chronic liver disorder worldwide, affecting approximately one-quarter to one-third of the adult population [1,2]. Rather than being a benign condition limited to hepatic lipid accumulation, MASLD represents a systemic metabolic disorder tightly linked to insulin resistance, chronic low-grade inflammation, mitochondrial dysfunction, and oxidative stress [3]. The disease encompasses a dynamic spectrum that may progress from simple steatosis to metabolic dysfunction-associated steatohepatitis (MASH), advanced fibrosis, cirrhosis, and hepatocellular carcinoma [4]. In addition, cardiovascular disease remains the leading cause of mortality in these patients, underscoring the systemic and multifactorial nature of the disorder [5].

Despite its growing global burden, therapeutic options specifically approved to halt or reverse MASLD progression remain limited. Most current interventions address metabolic risk factors without comprehensively targeting the complex inflammatory and signaling networks that drive disease progression through multiple interconnected pathways [6,7]. This limitation highlights the urgent need to identify agents capable of simultaneously modulating metabolic and inflammatory pathways.

Natural products continue to provide structurally diverse bioactive compounds with multitarget pharmacological potential [8]. Glycyrrhetinic acid, a pentacyclic triterpenoid derived from the hydrolysis of glycyrrhizin and found in the roots of *Glycyrrhiza* species, has attracted increasing attention due to its anti-inflammatory, antioxidant, and hepatoprotective properties [9,10]. Although both glycyrrhizin and glycyrrhetinic acid have been investigated in liver-related disorders, they should not be considered pharmacodynamically interchangeable, as glycyrrhetinic acid represents the primary intestinal and systemic metabolite with distinct molecular targets and biological effects. Previous experimental studies have reported that glycyrrhetinic acid can modulate nuclear factor kappa B (NF-κB) activation, reduce oxidative stress markers, and influence pro-inflammatory cytokine production in preclinical models of liver injury [11,12]. However, given the network-based pathophysiology of MASLD, a comprehensive systems-level characterization of its predicted molecular targets and interactions remains insufficiently explored.

Emerging evidence indicates that dysregulation of pro-inflammatory signaling pathways, particularly the NF-κB and Toll-like receptor (TLR) axes, plays critical roles in hepatic lipid accumulation, insulin resistance, and fibrogenic progression in MASLD, contributing to inflammatory amplification, hepatocellular injury, and disruption of lipid metabolism and adipogenic signaling. These proteins function as interconnected nodes within a broader molecular interactome that sustains disease progression [13,14].

Network pharmacology provides a systems-oriented framework for investigating complex diseases by integrating target prediction, PPI analysis, and network topological metrics to identify hub genes coordinating pathological processes [15,16]. Accordingly, the present study was designed not to demonstrate a definitive mechanism of action, but rather to generate mechanistic hypotheses and prioritize candidate therapeutic targets of glycyrrhetinic acid in MASLD using an integrated in silico workflow combining network pharmacology, molecular docking, and structural refinement.

## 2. Materials and Methods

### 2.1. Identification of Glycyrrhetinic Acid Targets

The chemical structure of glycyrrhetinic acid was retrieved from the PubChem database (National Center for Biotechnology Information), and the canonical SMILES string was used as the query input for target prediction analyses. Putative targets were predicted independently using two complementary platforms with distinct prediction algorithms: SwissTargetPrediction (accessed 29 January 2026) [17] and PharmMapper (Human Protein Targets Only Database, accessed 29 January 2026) [18]. For SwissTargetPrediction, the species was restricted to *Homo sapiens*, and only predicted targets with a probability score of ≥0.1 were retained. This cutoff was selected as an inclusive initial filter and is consistent with network pharmacology studies employing SwissTargetPrediction-based workflows [19]. This step yielded 89 predicted protein targets.

For PharmMapper, the same species restriction (*Homo sapiens*) was applied. The top 300 ranked targets were initially retained, and candidates were subsequently filtered based on a normalized fit score of ≥0.3, resulting in 263 predicted targets [20]. The outputs from both platforms were compiled and merged into a single dataset. Protein identifiers were converted to official gene symbols using the UniProt database, and duplicate entries were removed after identifier standardization, yielding 309 non-redundant glycyrrhetinic acid-associated targets.

To assess the robustness of database-specific threshold selection, additional sensitivity analyses were conducted. For SwissTargetPrediction, probability cutoffs of ≥0.1, ≥0.2, and ≥0.3 were compared while keeping the PharmMapper and MASLD filters constant. For PharmMapper, normalized fit score cutoffs of ≥0.1, ≥0.3, and ≥0.5 were compared, while maintaining fixed SwissTargetPrediction and MASLD criteria. In each scenario, the compound–target dataset was reconstructed, standardized, and compared based on the number of targets and overlap consistency. Detailed results are provided in the Supplementary Material.

### 2.2. Identification of MASLD-Related Genes

MASLD-associated genes were retrieved from the GeneCards database (accessed 1 February 2026) using the search term “Metabolic dysfunction-associated steatotic liver disease” [21]. A total of 338 genes were initially retrieved. To reduce weakly associated or potentially noisy entries, only genes with a relevance score of ≥45 were retained for downstream network construction. This threshold was applied as an operational stringency criterion to enrich for more strongly associated genes, rather than as a biologically definitive cutoff, since GeneCards relevance scores do not represent a universal biological scale [22,23]. After filtering, 173 MASLD-related genes were retained and subsequently standardized using the UniProt database. To further assess the robustness of this filtering strategy, additional sensitivity analyses were conducted using relevance score thresholds of ≥10, ≥30, and ≥45, while maintaining a fixed compound–target gene set. For each threshold, the number of retained MASLD-associated genes and the size of the intersecting gene set were compared. The corresponding results are reported in the Supplementary Material.

### 2.3. Identification of Candidate Therapeutic Targets and Construction of the Protein–Protein Interaction Network

The intersection between predicted glycyrrhetinic acid targets and MASLD-related genes was determined using Venny 2.1 “https://bioinfogp.cnb.csic.es/tools/venny/ (accessed on 5 February 2026)”. Overlapping genes were considered potential therapeutic targets for subsequent network and structural analyses. To determine whether the observed overlap between glycyrrhetinic acid-related targets and MASLD-associated genes was greater than expected by chance, a one-tailed hypergeometric test was performed. The background universe was defined as the set of 19,433 human protein-coding genes, according to the current GENCODE human release statistics “https://www.gencodegenes.org/human/stats.html (accessed on 29 January 2026)”. The analysis considered the number of glycyrrhetinic acid-related targets, the number of MASLD-associated genes, and the observed overlap between the two sets.

The common targets were imported into the STRING database “https://string-db.org/ (accessed on 10 February 2026)” [24] to construct a PPI network. The target organism was set to *Homo sapiens*, with a confidence score threshold of 0.5. Disconnected nodes in the network were removed to enhance clarity. The interaction network was exported in TSV format and visualized using Cytoscape (version 3.10.2) [25]. Network topology was analyzed using the CytoHubba plugin within Cytoscape [26]. Hub gene prioritization was performed using the MCC algorithm.

### 2.4. Functional Enrichment Analysis

GO enrichment (Biological Process, Molecular Function, and Cellular Component) and Kyoto Encyclopedia of Genes and Genomes (KEGG) pathway analysis were performed using the Metascape Bioinformatics Resources “https://metascape.org/gp/index.html (accessed on 17 February 2026)” [27]. A threshold of *p* < 0.05 was applied. Enrichment maps were generated to visualize the relationships among significantly enriched pathways.

### 2.5. Molecular Docking Analysis

#### 2.5.1. Protein Structure Retrieval and Preparation

Three-dimensional crystal structures of the target proteins were obtained from the Protein Data Bank “RCSB PDB; https://www.rcsb.org/ (accessed on 20 February 2026)”. Proteins were selected for docking based on their network relevance, biological involvement in inflammatory or metabolic pathways associated with MASLD, and the availability of experimentally resolved human structures suitable for small-molecule docking. Although several additional proteins emerged as relevant nodes in the network analysis, only AKT1, PPARγ, PPARα, and JAK2 were retained for docking-based structural prioritization in the revised workflow because of their biological relevance in MASLD and their suitability for downstream structural analysis. Based on these criteria, the following human protein structures were selected for molecular docking analysis: AKT1 (PDB ID: 4EKL), PPARγ (PDB ID: 4A4W), PPARα (PDB ID: 3VI8), and JAK2 (PDB ID: 2B7A).

The three-dimensional crystal structures of the target proteins were retrieved from the RCSB Protein Data Bank. The protein preparation was conducted through a dual-software workflow to ensure both biological relevance and computational stability. Initially, UCSF Chimera v1.16 was employed for structural curation [28]. This phase involved the systematic removal of non-catalytic water molecules, co-crystallized ligands, and heteroatoms to obtain the apo forms of the receptors. Subsequently, the prepared structures were imported into AutoDockTools (version 1.5.7) [29], where polar hydrogens were added and Kollman united atom charges were assigned. The search space for molecular docking was established by defining a grid box centered on the active-site coordinates for each target, with a spacing of 0.375 Å. Specifically, for AKT1 (PDB ID: 4EKL), the grid was centered at 28.032, 5.221, 10.891 with a box size of 20 × 20 × 20 Å. For PPARγ (4A4W), the search space was centered at −16.124, 17.081, −12.991 with dimensions of 30 × 30 × 30 Å, whereas for PPARα (3VI8), the grid box was centered at 11.247, 4.237, −7.449 with a size of 25 × 25 × 25 Å. In the case of JAK2 (2B7A), the center was established at 115.500, 60.021, 8.000 with a size of 22 × 22 × 22 Å. All parameters were saved in the PDBQT format required for the AutoDock Vina engine.

#### 2.5.2. Ligand Preparation

The two-dimensional structure of glycyrrhetinic acid was retrieved from the PubChem database in SDF format. Ligand preparation followed a systematic protocol in which glycyrrhetinic acid was maintained in its canonical neutral state. This approach was adopted to ensure a uniform baseline for competitive docking analysis across the targets. Structural refinement was initially performed using Avogadro v1.2.0, including the addition of all missing hydrogen atoms followed by energy minimization. Geometric optimization was carried out using the Merck Molecular Force Field (MMFF94) with a steepest descent algorithm to ensure a stable low-energy conformation. Subsequently, AutoDockTools was employed to assign Gasteiger partial charges and merge non-polar hydrogen atoms [29], preparing the ligand for molecular docking simulations.

#### 2.5.3. Docking Protocol

Molecular docking simulations were carried out using AutoDock Vina (version 1.2.3) [30]. The search space for molecular docking was systematically defined by centering the grid boxes on the coordinates of the co-crystallized ligand for each protein. In all simulations, grid dimensions were adjusted to fully encompass the target pocket while providing an adequate margin for ligand flexibility. The docking parameters included an exhaustiveness value of 32, 10 output poses, and a grid spacing of 0.375 Å. The conformation with the lowest binding free energy (kcal/mol) was selected as the representative binding mode for subsequent analysis.

To evaluate the reliability of the docking protocol, redocking experiments were performed for the co-crystallized ligands of the prioritized targets. For each protein, the native ligand was removed from the crystal structure and then redocked into the original binding site using the same docking parameters applied to glycyrrhetinic acid. Docking accuracy was assessed by calculating the root-mean-square deviation (RMSD) between the crystallographic pose and the redocked pose. An RMSD of ≤2.0 Å was considered indicative of acceptable reproduction of the experimental binding mode.

### 2.6. Molecular Dynamics Simulation

To further evaluate the stability of the docked complexes and refine target prioritization, 100 ns molecular dynamics (MD) simulations were performed for the glycyrrhetinic acid complexes with PPARγ (PDB ID: 4A4W), AKT1 (PDB ID: 4EKL), and JAK2. Each protein–ligand complex was prepared using the Desmond Molecular Dynamics System implemented in the Schrödinger suite. The systems were parameterized with the OPLS_2005 force field to describe bonded and non-bonded interactions. Each complex was solvated in an orthorhombic simulation box using the TIP3P explicit water model, maintaining a buffer distance of 10 Å between the solute and the box boundaries [31,32,33]. Counterions (Na^+^ and Cl^−^) were added to neutralize the systems, and the ionic concentration was adjusted to approximately 0.15 M to mimic physiological conditions. Before production runs, each system underwent the standard Desmond relaxation and equilibration protocol, including energy minimization and short equilibration stages to remove steric clashes. Production MD simulations were carried out under the NPT ensemble at 310 K and 1 atm for 100 ns. The trajectories were analyzed using the Simulation Interaction Diagram (SID) module to assess structural stability through protein and ligand root mean square deviation (RMSD), root-mean-square fluctuation (RMSF), protein secondary-structure elements (SSE), and radius of gyration (Rg). To complement the trajectory-based analyses, MM-GBSA rescoring was performed using the VSGB 2.0 solvation model implemented in Prime to account for solvent contributions to binding free energy calculations. Binding free energy was expressed as ΔG_bind_ (kcal/mol), and the main energetic components—including Coulombic, lipophilic, and van der Waals contributions—were extracted and averaged over the trajectory for comparative interpretation across the prioritized targets.

### 2.7. ADMET Properties Prediction

The pharmacokinetic profile and toxicity of glycyrrhetinic acid were evaluated using the admetSAR 2.0 server [34], a comprehensive tool for predicting absorption, distribution, metabolism, excretion, and toxicity properties based on chemical structure. The canonical SMILES (Simplified Molecular Input Line Entry System) string of the compound was used as the input for the predictive models. The analyzed parameters included physicochemical profile, drug-likeness, pharmacokinetics, and toxicity.

## 3. Results

### 3.1. Target Identification and Intersection Analysis

**Figure 1:**
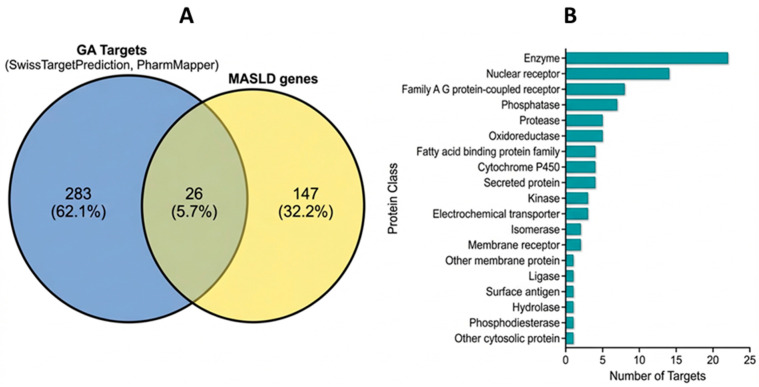
Identification of potential therapeutic targets of glycyrrhetinic acid in metabolic dysfunction-associated steatotic liver disease. (**A**) Venn diagram showing the intersection between glycyrrhetinic acid (GA) predicted targets (n = 309) and MASLD-related genes (n = 173). A total of 26 shared targets were identified. (**B**) Functional classification of the 26 intersecting targets based on protein class, highlighting a predominant representation of enzymes and nuclear receptors.

Potential targets of glycyrrhetinic acid were predicted using the SwissTargetPrediction and PharmMapper databases. After removing duplicate entries, a total of 309 unique targets were identified. MASLD-associated genes were retrieved from GeneCards, yielding 173 genes associated with disease pathophysiology. Intersection analysis between glycyrrhetinic acid targets and MASLD-related genes revealed 26 shared targets (Figure 1A). These common targets included key regulators involved in metabolic homeostasis, inflammatory signaling, and apoptosis, such as PPARγ, PPARα, JAK2, AKT1, TNFα, IL6, CASP3, and MMP9.

To determine whether this overlap was greater than expected by chance, a one-tailed hypergeometric test was performed using 19,433 human protein-coding genes as the background universe. The observed overlap of 26 shared targets between glycyrrhetinic acid-related targets and MASLD-associated genes was found to be significantly greater than expected by chance (*p* = 3.93 × 10^−18^), supporting the non-random nature of the observed intersection. Functional classification of the shared targets showed that they were mainly distributed among enzymes, nuclear receptors, kinases, and other protein classes (Figure 1B), suggesting that glycyrrhetinic acid may exert multitarget regulatory effects on metabolic and inflammatory pathways associated with MASLD.

### 3.2. Protein–Protein Interaction (PPI) Network Analysis

**Figure 2:**
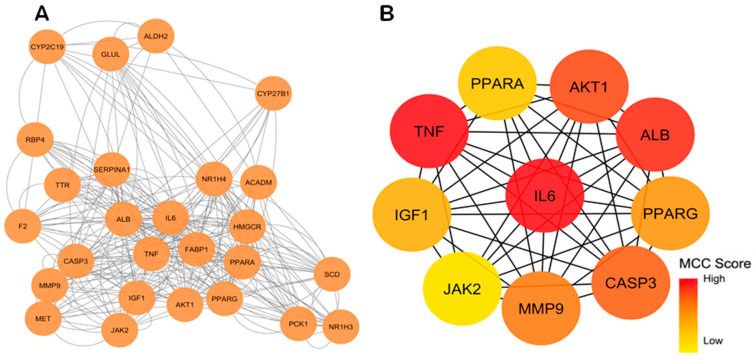
Protein–protein interaction network and identification of hub genes. (**A**) Protein–protein interaction (PPI) network of the intersecting targets between glycyrrhetinic acid and metabolic dysfunction-associated steatotic liver disease (MASLD), constructed using the STRING database and visualized in Cytoscape. Each node represents a protein, and edges represent predicted or experimentally supported protein–protein interactions. (**B**) Hub genes identified from the PPI network using the CytoHubba plugin based on the Maximal Clique Centrality (MCC) algorithm. Node color reflects the relative centrality score, with warmer colors indicating greater topological importance.

To explore the potential interactions among the intersecting targets associated with glycyrrhetinic acid and MASLD, a PPI network was constructed using the STRING database and visualized in Cytoscape (Figure 2A). The resulting network comprised several nodes interconnected by multiple interaction edges, indicating a dense interaction pattern among the targets. Notably, a group of proteins clustered at the center of the network exhibited a high degree of connectivity, suggesting a potential role in coordinating key biological processes such as inflammation, lipid metabolism, and metabolic regulation.

Within this network, several proteins displayed a high number of connections, with IL6, TNFα, AKT1, PPARγ, PPARα, and JAK2 standing out as biologically relevant nodes linked to inflammatory and metabolic regulation in MASLD. Among these, AKT1, PPARγ, PPARα, and JAK2 were prioritized for subsequent structural analysis because of their central network position and their established relevance to metabolic homeostasis, lipid regulation, and inflammatory signaling. To further identify the most relevant nodes within the network, topological analysis was performed using the CytoHubba plugin in Cytoscape based on the MCC algorithm. This analysis identified the top 10 hub genes with the highest centrality scores (Figure 2B). Among the highest-ranked nodes, IL6 and TNFα emerged as the most prominent central hubs, followed by AKT1 and PPARγ. Considering their biological relevance and structural suitability for small-molecule docking, AKT1, PPARγ, PPARα, and JAK2 were selected as the main targets for downstream structural prioritization.

### 3.3. Functional Enrichment Analysis and Compound–Target–Pathway Network Construction

**Figure 3:**
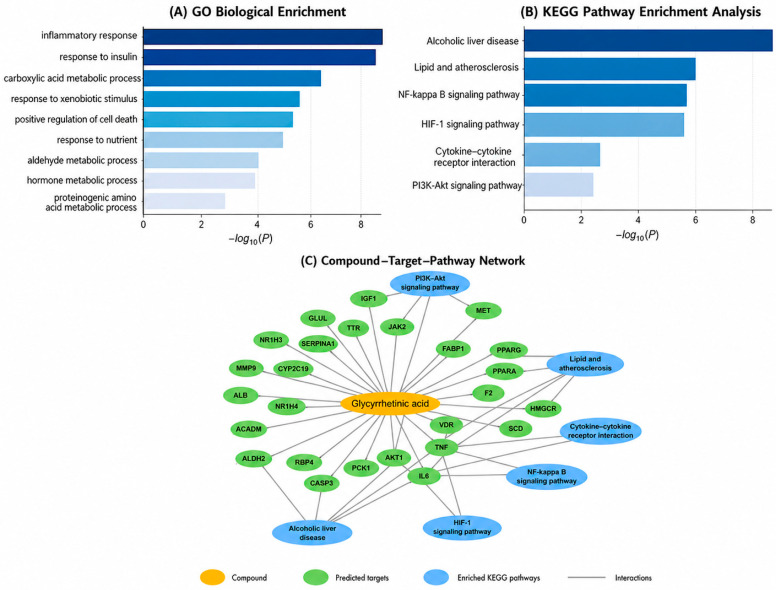
Functional enrichment analysis of the intersecting targets. (**A**) GO biological process enrichment analysis of the predicted targets. (**B**) KEGG pathway enrichment analysis highlighting signaling pathways associated with inflammation, metabolic regulation, and liver disease. (**C**) Compound–target–pathway network illustrating the interactions between glycyrrhetinic acid, predicted targets, and enriched KEGG pathways. The orange node represents glycyrrhetinic acid, green nodes represent predicted protein targets, and blue nodes represent enriched KEGG.

To further investigate the biological functions associated with the intersecting targets, GO biological process enrichment analysis was performed using the Metascape platform. The results revealed that the predicted targets were significantly enriched in biological processes mainly related to inflammation, metabolic regulation, and cellular stress responses (Figure 3A).

The most prominent enrichment was observed in inflammatory response, insulin signaling, and carboxylic acid metabolic processes. Additional enriched biological processes included response to xenobiotic stimulus, positive regulation of cell death, and response to nutrients, suggesting that these proteins may contribute to cellular adaptation under metabolic stress conditions. Other enriched processes, such as aldehyde metabolic process, hormone metabolic process, and proteinogenic amino acid metabolic process, further support the involvement of these targets in metabolic homeostasis and liver-associated biochemical functions.

To further explore the signaling pathways involved, KEGG pathway enrichment analysis was conducted (Figure 3B). The analysis highlighted several pathways associated with hepatic injury, inflammation, and metabolic dysregulation. Among the most significantly enriched pathways were alcoholic liver disease, lipid and atherosclerosis, the NF-κB signaling pathway, and the HIF-1 signaling pathway. In addition, cytokine–cytokine receptor interaction and PI3K–Akt signaling pathway were also enriched.

Overall, the enrichment results indicate that the predicted targets are mainly involved in interconnected networks related to inflammation, lipid metabolism, and metabolic regulation, which are key processes underlying MASLD pathogenesis. Additionally, to visualize the relationships between glycyrrhetinic acid, the predicted targets, and the enriched pathways, a compound–target–pathway network was constructed using Cytoscape (Figure 3C). The network highlights the multi-target and multi-pathway characteristics of glycyrrhetinic acid, suggesting that its potential relevance in MASLD may involve coordinated regulation of inflammatory and metabolic signaling pathways.

### 3.4. Molecular Docking Study

#### 3.4.1. Docking Protocol Validation

To evaluate the reliability of the docking protocol, redocking experiments were performed for the co-crystallized ligands of the prioritized targets. The native ligands were removed from the crystal structures and subsequently redocked into the same binding region using the docking parameters applied to glycyrrhetinic acid. The resulting redocked poses reproduced the experimental binding modes with RMSD values of 1.676 Å for AKT1 (4EKL), 1.146 Å for PPARγ (4A4W), 0.584 Å for PPARα (3VI8), and 0.302 Å for JAK2 (2B7A). All values were below the commonly accepted validation threshold of 2.0 Å, supporting the reliability of the docking protocol for the selected targets.

#### 3.4.2. Molecular Docking Analysis of Glycyrrhetinic Acid with MASLD-Related Targets

**Figure 4:**
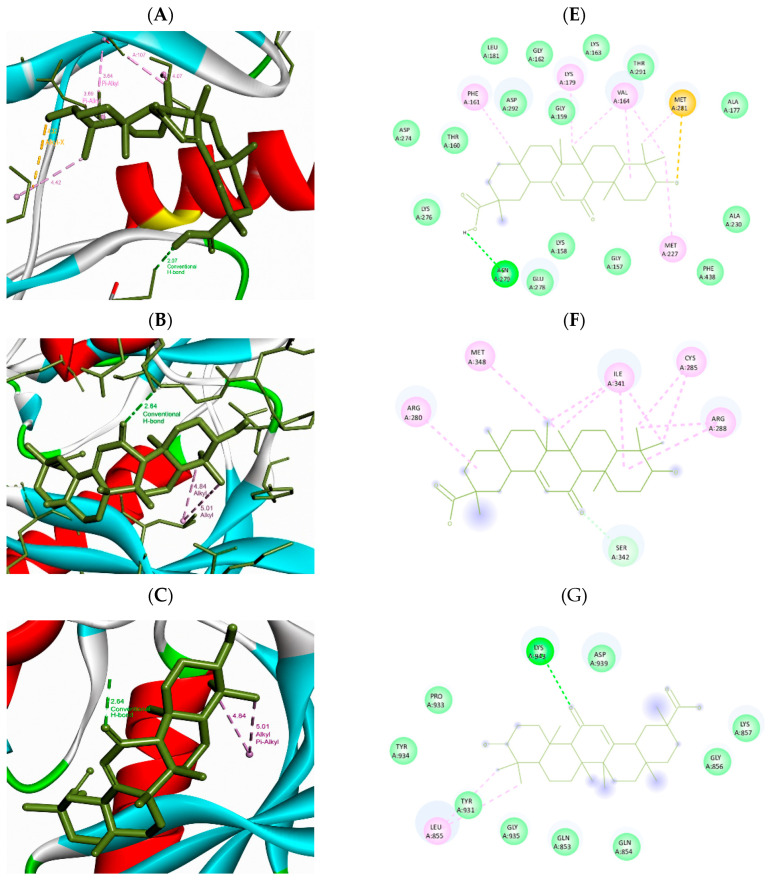

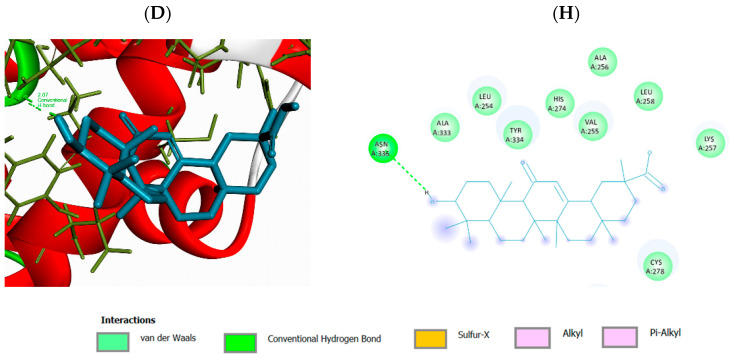
Two and three-dimensional binding poses of glycyrrhetinic acid within the active sites of selected MASLD-related targets (**A**,**E**) AKT1, (**B**,**F**) PPARγ, (**C**,**G**) JAK2 and (**D**,**H**) PPARα.

Molecular docking analysis was performed for the structurally prioritized targets AKT1, PPARγ, PPARα, and JAK2. Among the analyzed proteins, PPARγ showed the most favorable predicted binding affinity for glycyrrhetinic acid (−7.200 ± 0.017 kcal/mol), followed by AKT1 (−7.012 ± 0.009 kcal/mol) and JAK2 (−6.063 ± 0.176 kcal/mol). PPARα showed the lowest predicted binding affinity among the analyzed targets (−3.776 ± 0.038 kcal/mol). The main interacting residues identified for AKT1 were ASN279, PHE161, MET227, VAL164 and MET281, which formed hydrogen-bond and hydrophobic contacts. The interaction profiles of the other prioritized complexes are summarized in Table 1. Detailed three-dimensional binding poses and two-dimensional interaction diagrams of the prioritized complexes are presented in Figure 4.

**Figure 5:**
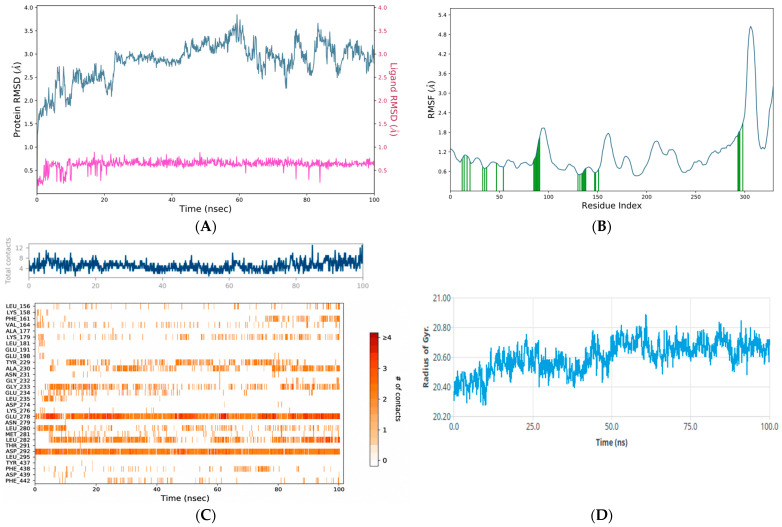
(**A**) Structural stability of the AKT1-glycyrrhetinic acid complex during the molecular dynamics simulation. The left *y*-axis represents protein RMSD, whereas the right *y*-axis represents ligand RMSD. The darker color represents ligand RMSD, whereas the lighter blue color represents protein RMSD. This panel illustrates the overall structural stability of the target protein, including conformational stability and the stability of the ligand within the binding interface. (**B**) The local flexibility profile of the receptor protein residues in the AKT1-glycyrrhetinic acid complex. The x-axis indicates the index of the residues, and the y-axis indicates the magnitude of the fluctuations in angstroms (Å). The red vertical lines indicate residues interacting with the ligand. (**C**) The time-dependent evolution of the number of hydrogen bonds between the complex and the receptor active site throughout the simulation trajectory. The strength and persistence of the polar interaction network are shown in a heatmap plot; a consistently higher number of hydrogen bonds is associated with stronger binding affinity and greater complex stability. (**D**) Radius of gyration analysis of the AKT1 complex during the simulation.

Detailed three-dimensional binding poses and two-dimensional interaction diagrams of the prioritized complexes are presented in Figure 4 and Figure 5. The main interacting residues, interaction types, and key contact distances for these complexes should be considered alongside the docking scores to support target prioritization.

### 3.5. Molecular Dynamics Analysis

**Figure 6:**
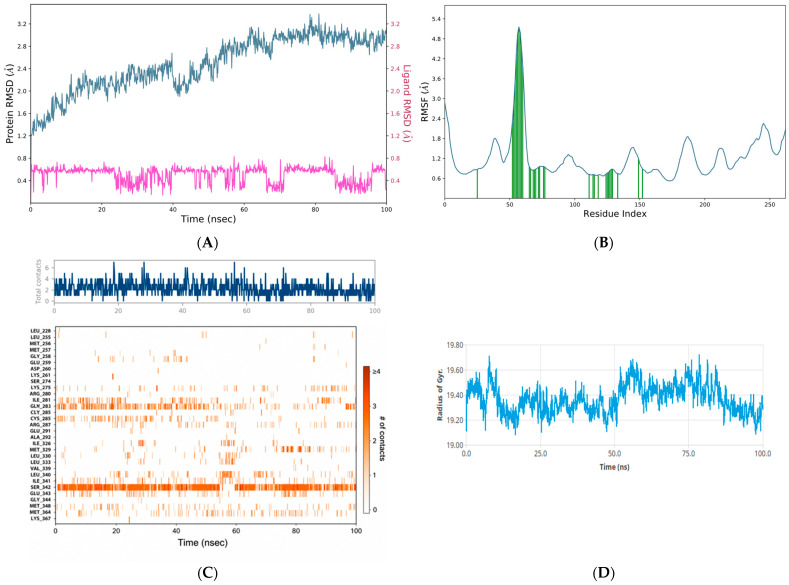
(**A**) RMSD profile showing the structural stability of the PPARγ-glycyrrhetinic acid complex during the molecular dynamics simulation. The left y-axis represents protein RMSD, whereas the right y-axis represents ligand RMSD. The darker color represents ligand RMSD, whereas the lighter blue color represents protein RMSD. This panel illustrates the overall structural stability of the target protein, including conformational stability and the stability of the ligand within the binding interface. (**B**) RMSF profile showing the local flexibility of the receptor protein residues in the complex. The x-axis indicates the residue index, and the y-axis indicates the magnitude of the fluctuations in angstroms (Å). The red vertical lines indicate residues interacting with the ligand. (**C**) The time-dependent evolution of the number of hydrogen bonds between the PPARγ-glycyrrhetinic acid complex and the receptor active site throughout the simulation trajectory. The strength and persistence of the polar interaction network are shown in a heatmap plot; a consistently higher number of hydrogen bonds is associated with stronger binding affinity and greater complex stability. (**D**) Radius of gyration analysis of the PPARγ complex during the simulation.

**Figure 7:**
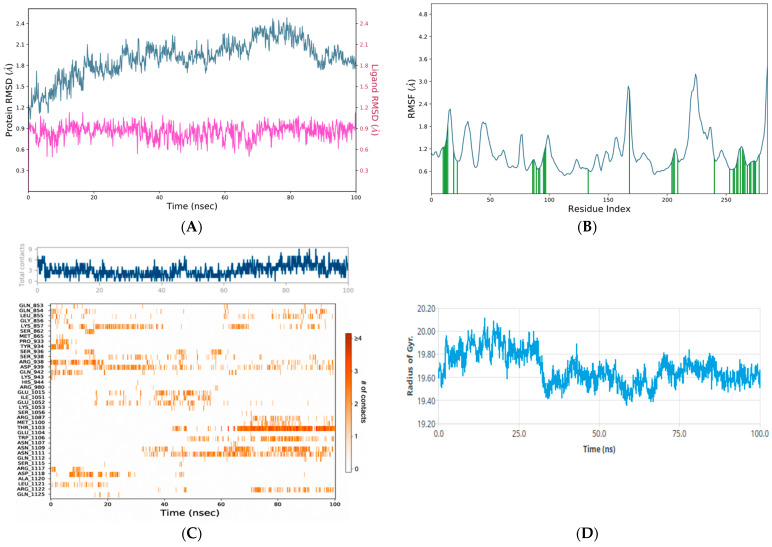
(**A**) RMSD profile showing the structural stability of the JAK2-glycyrrhetinic acid complex during the molecular dynamics simulation. The left y-axis represents protein RMSD, whereas the right y-axis represents ligand RMSD. The darker color represents ligand RMSD, whereas the lighter blue color represents protein RMSD. This panel illustrates the overall structural stability of the target protein, including conformational stability and the stability of the ligand within the binding interface. (**B**) RMSF profile showing the local flexibility of the receptor protein residues in the JAK2-glycyrrhetinic acid complex. The x-axis indicates the residue index, and the y-axis indicates the magnitude of the fluctuations in angstroms (Å). The red vertical lines indicate residues interacting with the ligand. (**C**) The time-dependent evolution of the number of hydrogen bonds between the JAK2-glycyrrhetinic acid complex and the receptor active site throughout the simulation trajectory. The strength and persistence of the polar interaction network are shown in a heatmap plot; a consistently higher number of hydrogen bonds is associated with stronger binding affinity and greater complex stability. (**D**) Radius of gyration analysis of the JAK2 complex during the simulation.

To refine the docking-based target selection, 100 ns MD simulations were performed for glycyrrhetinic acid complexes with PPARγ (PDB: 4A4W), AKT1 (PDB: 4EKL), and JAK2 (Figure 5, Figure 6 and Figure 7). Overall, all three systems remained structurally stable throughout the simulation period; however, clear differences were observed in their dynamic behavior and binding free energy profiles. Backbone RMSD analysis showed that the PPARγ–glycyrrhetinic acid complex (Figure 6A) reached equilibrium early and remained stable over the trajectory, with protein RMSD values predominantly within 2.0–3.1 Å. The ligand RMSD remained exceptionally low (~0.3–0.8 Å), indicating a highly stable binding mode. The AKT1–glycyrrhetinic acid complex also showed sustained ligand retention, although with somewhat greater receptor mobility (protein RMSD: 2.5–3.4 Å) (Figure 5A). Despite this broader conformational adjustment, ligand RMSD remained relatively low (~0.5–0.8 Å). Among the evaluated systems, the JAK2–glycyrrhetinic acid complex exhibited the lowest overall backbone deviation (~1.3–2.4 Å), with ligand RMSD within ~0.6–1.0 Å, consistent with persistent association throughout the simulation (Figure 7A).

Residue-level flexibility, assessed by RMSF, showed that higher fluctuations were concentrated primarily in terminal and loop regions, while structured domains remained stable, indicating preservation of the overall fold. This was supported by secondary-structure analysis (SSE). PPARγ displayed the highest proportion of ordered structure (53.34% total SSE) (Figure 6B), followed by JAK2 (44.16%) (Figure 7B) and AKT1 (38.34%) (Figure 5B). None of the complexes underwent major secondary-structure disruption upon ligand binding.

#### 3.5.1. Hydrogen-Bond Analysis

Hydrogen-bond analysis was performed to evaluate the strength and persistence of intermolecular interactions within the AKT1, PPARγ, and JAK2 complexes (Figure 5C, Figure 6C and Figure 7C). Timeline analysis revealed consistent hydrogen-bond formation throughout the simulation. Specifically, an average of approximately 10–12 hydrogen bonds was maintained across the trajectory for the AKT1 and JAK2 complexes, whereas an average of approximately 6–8 hydrogen bonds was maintained for the PPARγ-glycyrrhetinic acid complex. The heatmap representation further confirmed the persistence of these polar interactions, indicating a stable interaction network in all cases.

#### 3.5.2. Radius of Gyration Analysis

Protein compactness was assessed through radius of gyration (Rg) analysis. The PPARγ-glycyrrhetinic acid complex showed the narrowest Rg distribution, fluctuating within 19.1–19.7 Å (Figure 6D). After a brief equilibration phase, the system remained, particularly stable between 20 and 70 ns, with values around 19.3–19.5 Å. The JAK2-glycyrrhetinic acid complex exhibited Rg values between 19.3 and 20.1 Å (Figure 7D). A slightly expanded conformation was observed during the initial stage, followed by a decrease around 30–40 ns and subsequent stabilization around 19.5–19.7 Å. In the AKT1-glycyrrhetinic acid system, Rg values ranged from 20.3 to 20.9 Å (Figure 5D), indicating slightly greater variability and a somewhat less compact conformation than that observed for PPARγ. After an initial increase, the system stabilized around 20.5–20.7 Å, without evidence of major unfolding or structural disruption.

#### 3.5.3. MM-GBSA Rescoring

To address the limitations of the initial docking scores and provide a more robust thermodynamic ranking, Prime MM-GBSA rescoring was performed using the 100 ns trajectories. Among the three evaluated targets, PPARγ exhibited the most favorable binding free energy, with an average ΔG_bind of −100.871 kcal/mol. The main stabilizing contributions were lipophilic interactions (−62.74 kcal/mol) and van der Waals interactions (−47.42 kcal/mol). Notably, the Coulombic term (ΔG_coulomb_ = −8.84 kcal/mol) provided an additional electrostatic contribution that reinforced complex stability.

The AKT1–glycyrrhetinic acid complex showed the second-most favorable profile, with an average ΔG_bind_ of −70.18 kcal/mol. As observed for PPARγ, the dominant favorable contributions arose from lipophilic (−39.75 kcal/mol) and van der Waals (−45.60 kcal/mol) interactions, while the Coulombic (−8.66 kcal/mol) and hydrogen-bond (−1.18 kcal/mol) components were comparatively smaller. By contrast, the JAK2–glycyrrhetinic acid complex yielded a substantially less favorable average binding free energy (ΔG_bind = −26.85 kcal/mol). This weaker energetic profile was accompanied by lower lipophilic (−14.78 kcal/mol) and van der Waals (−22.40 kcal/mol) contributions than those observed for PPARγ and AKT1.

### 3.6. ADMET Profile of Glycyrrhetinic Acid

An in silico ADMET assessment was performed for glycyrrhetinic acid in order to evaluate its drug-likeness and translational suitability (Table 2). The compound showed a molecular weight of 470.69 g/mol, with 4 hydrogen-bond donors, 2 hydrogen-bond acceptors, a topological polar surface area (TPSA) of 74.60 Å^2^, and 1 rotatable bond, indicating a relatively rigid scaffold with moderate polarity. Its lipophilicity (MLOGP = 4.87) suggests a markedly hydrophobic character, which may favor hydrophobic contacts within protein binding pockets but may also impose limitations on aqueous behavior and systemic disposition. Drug-likeness analysis showed that glycyrrhetinic acid presented only one Lipinski violation and no PAINS alerts, supporting the absence of obvious structural liabilities associated with nonspecific assay interference. These features are consistent with a generally acceptable small-molecule profile from a medicinal chemistry perspective, although not necessarily an optimal developability profile.

The pharmacokinetic predictions revealed a mixed profile. Gastrointestinal absorption was predicted to be high, suggesting that the compound may possess favorable membrane permeability. However, glycyrrhetinic acid was also predicted to be a P-glycoprotein substrate, which may reduce net intestinal absorption and contribute to variability in systemic exposure. In addition, the compound was predicted to be non-permeable to the blood–brain barrier, indicating limited central nervous system distribution. Regarding metabolism-related properties, glycyrrhetinic acid was predicted to be non-inhibitory toward CYP1A2, CYP2D6, and CYP3A4, which may indicate a relatively low risk of inhibition-based drug–drug interactions for these isoforms. At the same time, the prediction that it may act as a CYP3A4 substrate suggests that metabolic clearance through this pathway could influence its systemic persistence and bioavailability.

The toxicity profile also suggested a mixed safety signal. The predicted probabilities for AMES toxicity and hERG I inhibition were low (5.6% and 1.6%, respectively), indicating a limited likelihood of mutagenicity and a low risk for one major component of cardiotoxic liability. By contrast, the probability of hERG II inhibition was considerably higher (61.2%), suggesting that potential cardiac safety concerns cannot be excluded and should be considered in the interpretation of its translational potential.

## 4. Discussion

MASLD is a complex disorder characterized by the interplay between lipid accumulation, chronic inflammation, oxidative stress, and dysregulated metabolic signaling [35]. These interconnected mechanisms involve multiple molecular pathways, making MASLD particularly suitable for the exploration of multitarget-oriented pharmacological hypotheses. In this context, the present network pharmacology and molecular docking analyses do not demonstrate therapeutic activity or pathway modulation, but rather provide an in silico framework to prioritize potential targets and pathways through which glycyrrhetinic acid may exert relevant effects in MASLD.

The intersection analysis between predicted glycyrrhetinic acid targets and MASLD-associated genes identified 26 shared targets, several of which are central regulators of metabolic and inflammatory signaling, including AKT1, PPARγ, PPARα, TNFα, IL6, JAK2, CASP3, and MMP9. These proteins are known to participate in hepatic lipid metabolism, inflammatory responses, and cellular stress pathways linked to disease progression from steatosis to steatohepatitis and fibrosis [1,35]. Accordingly, the identification of these overlapping targets should be interpreted as a target-prioritization step rather than as evidence of direct biological regulation by glycyrrhetinic acid.

The PPI network analysis further highlighted IL6, TNFα, AKT1, and PPARγ as central hub genes with high connectivity within the interaction network. This observation is consistent with previous studies showing that inflammatory cytokines and metabolic regulators act as key nodes linking metabolic dysregulation with hepatic inflammation. In particular, TNFα and IL-6 are widely recognized mediators of liver inflammation and insulin resistance, contributing to hepatocyte injury and the progression of fatty liver disease [36,37]. Elevated levels of these cytokines have been consistently associated with disease severity and progression toward steatohepatitis and fibrosis [36,37,38]. However, their identification as hubs in the present network does not establish that they are directly modulated by glycyrrhetinic acid; rather, it indicates that they occupy central positions in the shared interactome and may therefore merit further experimental evaluation.

Functional enrichment analysis further supported the biological plausibility of the shared target set. Gene Ontology results indicated that the intersecting targets were mainly associated with biological processes such as inflammatory response, response to insulin, and carboxylic acid metabolic process, all of which are relevant to MASLD pathophysiology. Similarly, KEGG pathway analysis highlighted NF-κB signaling, HIF-1 signaling, PI3K–Akt signaling, cytokine–cytokine receptor interaction, and lipid and atherosclerosis pathways. Activation of NF-κB promotes hepatic inflammation and cytokine production, whereas dysregulation of PI3K–Akt signaling contributes to insulin resistance and hepatic lipid accumulation [39,40]. In addition, hypoxia-related signaling mediated by HIF-1α has been implicated in hepatic steatosis and inflammatory responses in fatty liver disease. Nevertheless, enrichment of these pathways should be interpreted cautiously, as KEGG categories are often broad and may reflect shared inflammatory or metabolic genes rather than disease-specific mechanisms uniquely attributable to MASLD.

The compound–target–pathway network further illustrated the predicted multitarget profile of glycyrrhetinic acid within the selected in silico framework. Such a profile is conceptually relevant in metabolic diseases, where metabolic and inflammatory processes are tightly interconnected. Previous pharmacological studies have reported anti-inflammatory, antioxidant, and hepatoprotective activities of glycyrrhetinic acid, supporting its relevance as a bioactive compound of interest in liver-related disorders [9,10,41]. However, the present study does not experimentally verify these effects in MASLD; therefore, its contribution lies in mechanistic hypothesis generation rather than biological confirmation.

The molecular docking analysis provided comparative structural support for some of the predicted target–compound pairs. Among the evaluated proteins, AKT1 and PPARγ showed the most favorable docking scores. These results suggest that glycyrrhetinic acid may exhibit greater predicted binding affinity toward proteins involved in metabolic regulation and lipid homeostasis, particularly AKT1 and PPARγ. AKT1 is a central component of the PI3K–Akt signaling pathway, which regulates glucose metabolism, insulin signaling, and lipid homeostasis in hepatocytes [38,39,42,43]. Dysregulation of AKT1 signaling has been implicated in hepatic insulin resistance and steatosis, indicating that this node is highly relevant in MASLD. However, docking scores should be interpreted only as approximate indicators of structural compatibility and not as evidence of functional modulation.

To refine the preliminary docking-based ranking, the top-prioritized complexes were further evaluated by 100 ns molecular dynamics simulations and MM-GBSA rescoring. This integrated approach provided additional information on structural stability, residue-level flexibility, and relative binding energetics. RMSF analysis showed that flexibility was mainly localized to loop and terminal regions, whereas structured domains remained comparatively stable across all systems. Secondary structure analysis further supported this observation, with PPARγ displaying the highest proportion of ordered structural elements. MM-GBSA rescoring prioritized PPARγ as the most favorable target among the evaluated complexes, with a ΔG_bind_ of −100.871 kcal/mol. This favorable profile was largely driven by lipophilic and van der Waals contributions, consistent with the hydrophobic nature of glycyrrhetinic acid as a pentacyclic triterpenoid. Previous studies have reported that glycyrrhetinic acid and related compounds can modulate PPARγ-associated pathways involved in lipid metabolism and inflammation, supporting the disease relevance of this target in metabolic liver disorders [44,45].

The AKT1–glycyrrhetinic acid complex showed the second-most favorable energetic profile (ΔG_bind_ = −70.18 kcal/mol), supporting its consideration as a structurally plausible secondary target. AKT1 is a central component of the PI3K–Akt signaling pathway, which regulates insulin signaling, lipid metabolism, and hepatocellular survival [46]. Dysregulation of this pathway has been strongly associated with insulin resistance and hepatic steatosis, highlighting its relevance in MASLD pathophysiology. Although the present computational data support structural compatibility between glycyrrhetinic acid and AKT1, they do not demonstrate direct inhibition or functional modulation of the kinase.

In contrast, the JAK2–glycyrrhetinic acid complex exhibited a substantially less favorable binding free energy (ΔG_bind_ = −26.85 kcal/mol), despite showing stable structural behavior during MD simulations. JAK2 plays an important role in cytokine signaling and inflammation, but the present results place it at a lower level of structural plausibility relative to PPARγ and AKT1 under the current computational conditions. Therefore, any potential involvement of JAK/STAT signaling in glycyrrhetinic acid activity may be indirect or context-dependent rather than driven by direct high-affinity binding. Taken together, the integration of network pharmacology, docking, molecular dynamics, and MM-GBSA rescoring does not demonstrate that glycyrrhetinic acid exerts therapeutic effects in MASLD, but it does support the prioritization of a subset of targets, particularly PPARγ and AKT1 as plausible candidates for further investigation. In this sense, the present findings are better understood as a mechanistic, hypothesis-generating framework that may guide future experimental studies in cellular and in vivo models of MASLD.

The enrichment of the alcoholic liver disease pathway should not be interpreted as evidence that MASLD and alcohol-related liver disease are equivalent entities. Rather, this finding likely reflects the fact that both conditions share core pathogenic processes, including inflammatory signaling, oxidative stress, hepatocellular injury, and fibrogenic responses. In KEGG, several genes identified in our intersected set, such as TNFα, IL6, and AKT1-related signaling nodes, participate in multiple liver disease maps, including alcoholic liver disease, non-alcoholic fatty liver disease, and insulin resistance. Therefore, enrichment of this pathway most likely captures shared inflammatory–metabolic biology rather than an alcohol-specific etiology. At the same time, we acknowledge that this result also indicates that the present gene set is enriched for broadly relevant hepatic injury pathways and is not fully disease-exclusive to MASLD. Accordingly, this pathway was interpreted as supportive of mechanistic overlap, but not as evidence against the disease specificity of MASLD as a distinct clinic-pathological entity [47,48].

Notably, relatively few studies have focused specifically on glycyrrhetinic acid in MASLD/NAFLD or closely related metabolic liver disease contexts. One relevant example is the work of Fan et al. [49], who showed that glycyrrhetinic acid ameliorated NAFLD-associated macrophage dysfunction by regulating autophagic flux, supporting its biological relevance in fatty liver disease, although not within a network pharmacology framework. By contrast, a larger body of related evidence involves structurally related compounds or derivatives, particularly diammonium glycyrrhizinate, as well as broader Glycyrrhiza-derived interventions. For instance, Qin et al. [50] used a network pharmacology and molecular docking strategy to identify anti-liver injury targets of diammonium glycyrrhizinate, highlighting inflammatory and injury-related hubs such as TNFα, CASP3, PTGS2, ALB, STAT3, and TLR4. Likewise, Gao et al. [51] demonstrated that diammonium glycyrrhizinate mitigates liver injury through anti-inflammatory and anti-apoptotic mechanisms, further supporting the relevance of *glycyrrhizin*-related scaffolds in hepatic disease. Taken together, these studies indicate that the literature directly centered on glycyrrhetinic acid itself remains comparatively limited, whereas more abundant evidence is available for related analogs or *Glycyrrhiza*-derived compounds. In this context, the present findings are broadly consistent with the recurrent identification of inflammation- and metabolism-related targets in liver disease. They also add value by providing a glycyrrhetinic acid-specific, MASLD-focused network pharmacology analysis further refined by docking, molecular dynamics, MM-GBSA rescoring, and ADMET evaluation.

From a translational perspective, the physicochemical and ADMET profile of glycyrrhetinic acid further refines target plausibility. Its relatively high lipophilicity and poor aqueous solubility may favor partitioning into lipid-rich intracellular compartments, supporting the plausibility of interactions with nuclear and membrane-associated targets such as PPARγ, PPARα, and AKT1. Conversely, these same properties may reduce the likelihood of meaningful in vivo interactions with hydrophilic extracellular mediators such as IL-6 or TNFα, despite their biological relevance and network centrality. Thus, integrating ADMET considerations with structural compatibility further narrows the most translationally plausible targets to intracellular metabolic regulators, particularly PPARγ and AKT1, whereas PPARα remains biologically relevant but less strongly supported after post-docking refinement [52].

This study has several limitations that should be considered when interpreting the findings. Because the analysis was based on public databases and in silico prediction tools, the results may be influenced by database curation, coverage, and algorithm-specific bias. In addition, target selection and network construction depended on predefined filtering criteria, which may have affected candidate prioritization. The PPI and enrichment analyses provide a systems-level view of potential associations, but do not establish causal or disease-specific mechanisms. Likewise, molecular docking offers only a static estimate of potential binding compatibility and does not demonstrate binding stability or functional activity. Although molecular dynamics and MM-GBSA analyses provide a more refined structural and energetic perspective, they still do not establish biological efficacy. Future studies should therefore integrate transcriptomic or proteomic data, functional assays, and disease-relevant experimental models to validate whether the prioritized targets identified here are truly involved in the biological effects of glycyrrhetinic acid in MASLD. An additional methodological limitation is that crystallographic water molecules were removed during receptor preparation. Although this is common in standardized docking workflows, it may omit conserved active-site waters that contribute to ligand recognition through water-mediated hydrogen-bond networks.

## 5. Conclusions

In conclusion, the present study establishes a systematic computational framework to explore the molecular basis of glycyrrhetinic acid in the context of MASLD. By integrating network pharmacology, molecular docking, molecular dynamics simulations, and MM-GBSA rescoring, we identified a robust interactome associated with inflammatory and metabolic regulation. Among the prioritized targets, PPARγ emerged as the most consistent candidate, with AKT1 also showing a high-confidence interaction profile after dynamic refinement, while JAK2 appeared less supported by the energetic analysis.

These findings suggest that glycyrrhetinic acid may interact with molecular networks relevant to lipid homeostasis, inflammatory signaling, and hepatocellular injury, thereby providing a mechanistically coherent basis for further investigation. However, the present results remain predictive and should not be interpreted as evidence of direct therapeutic efficacy or validated target modulation. In particular, the ADMET profiling indicates that, despite favorable target engagement in silico, the translational potential of glycyrrhetinic acid may be constrained by pharmacokinetic and safety-related liabilities.

Ultimately, this work positions glycyrrhetinic acid as a biologically relevant scaffold and a high-confidence, hypothesis-generating molecule for metabolic research. Subsequent experimental validation using target-specific biochemical assays and disease-relevant in vitro or in vivo MASLD models will be essential to corroborate these predicted interactions and to fully elucidate their functional significance in modulating hepatic lipid metabolism and inflammation.

## Figures and Tables

**Table 1:** Binding affinities and molecular interaction residues of glycyrrhetinic acid within MASLD-related hub protein pockets.

Target	PDB ID	Co-Crystallized Ligand	Redocking-RMSD (Å)	GA Binding Affinity (kcal/mol)	Pairwise Pose RMSD (Å)	Key Interacting Residues
AKT1	4EKL	0RF	1.676	−7.012 ± 0.009	0.160 ± 0.071	ASN279 (H-bond) PHE161, MET227, VAL164 (Pi-Alkyl) MET281 (Sulfur)
PPARγ	4A4W	YFB	0.918	−7.200 ± 0.017	0.341 ± 0.115	LYS943 (H-bond) LEU855 (Alkyl)
PPARα	3VI8	13M	0.584	−3.776 ± 0.038	0.338 ± 0.261	ASN336 (H-bond)
JAK2	2B7A	IZA	0.302	−6.063 ± 0.176	0.222 ± 0.103	ASN336 (H-bond)

0RF: Ipatasertib; YFB: AMORFRUTIN B; 13M: (2S)-2-(4-methoxy-3-{[(pyren-1-ylcarbonyl)amino]methyl}benzyl)butanoic acid; IZA: Pyridone 6; GA: glycyrrhetinic acid.

**Table 2:** In silico ADMET-related profile of glycyrrhetinic acid.

Domain	Parameter	Predicted/Observed Value
Physicochemical profile	Molecular weight	470.69
	Hydrogen-bond donors	4
	Hydrogen-bond acceptors	2
	Topological polar surface area (TPSA, Å^2^)	74.60
	Lipophilicity (MLOGP)	4.87
	Rotatable bonds	1
Drug-likeness	Lipinski violations (n)	1
	PAINS alerts	0
Pharmacokinetics	Gastrointestinal absorption	High
	Blood–brain barrier permeability	No
	P-gp substrate	Yes
	CYP1A2 inhibitor	No
	CYP2D6 inhibitor	No
	CYP3A4 inhibitor	No
	CYP1A2 substrate	No
	CYP2D6 substrate	No
	CYP3A4 substrate	Yes
Toxicity/safety	AMES toxicity	5.6%
	hERG I inhibition	1.6%
	hERG II inhibition	61.2%

## Data Availability

The data are contained within the article.

## Supplementary Materials

The following supporting information can be downloaded at: https://www.mdpi.com/article/10.3390/metabo16050301/s1, Table S1: SwissTargetPrediction cut-off points; Table S2: PharmaMapper cut-off points; Table S3: GeneCard cut-off points.
