# Supplementary material for: Network Pharmacology Analysis of Glycyrrhetinic Acid in Metabolic Dysfunction-Associated Steatotic Liver Disease
Source: Metabolites. 2026 Apr 29;16(5):301. doi: 10.3390/metabo16050301 (PMC13208598; doi:10.3390/metabo16050301)
Supplement: Supplementary file 1 [file metabolites-16-00301-s001.zip › metabolites-4218415-supplementary.pdf]

Table S1 SwissTargetPrediction cut-off points

**STP cutoff**	**PM fixed**	**MASLD fixed**	**Compound-target set size**	**Shared targets (n)**	Overlapping targets retained
≥0.1	top 300	≥45	309	26	ALB, TTR, PPARG, MET, PPARA, F2, RBP4, NR1H4, AKT1, NR1H3, IGF1, HMGCR, CASP3, SERPINA1, VDR, PCK1, ALDH2, JAK2, ACADM, MMP9, FABP1, SCD, TNF, CYP2C19, IL6, GLUL
≥0.2	top 300	≥45	237	26	ALB, TTR, PPARG, MET, PPARA, F2, RBP4, NR1H4, AKT1, NR1H3, IGF1, HMGCR, CASP3, SERPINA1, VDR, PCK1, ALDH2, JAK2, ACADM, MMP9, FABP1, SCD, TNF, CYP2C19, IL6, GLUL
≥0.3	top 300	≥45	229	20	ALB, TTR, PPARG, MET, PPARA, F2, RBP4, NR1H4, AKT1, NR1H3, IGF1, HMGCR, CASP3, SERPINA1, VDR, PCK1, ALDH2, JAK2, ACADM, MMP9

Table S2 PharmaMapper cut-off points

**STP cutoff**	**PM fixed**	**GeneCard**	**Compound-target set size**	**Shared targets (n)**	Overlapping targets retained
≥0.1	≥0.1	≥45	347		ALB, TTR, PPARG, MET, PPARA, F2, RBP4, NR1H4, AKT1, NR1H3, IGF1, HMGCR, CASP3, SERPINA1, VDR, PCK1, ALDH2, JAK2, ACADM, MMP9, FABP1, SCD, TNF, CYP2C19, IL6, GLUL
≥0.1	≥0.3	≥45	309	26	ALB, TTR, PPARG, MET, PPARA, F2, RBP4, NR1H4, AKT1, NR1H3, IGF1, HMGCR, CASP3, SERPINA1, VDR, PCK1, ALDH2, JAK2, ACADM, MMP9, FABP1, SCD, TNF, CYP2C19, IL6, GLUL
≥0.2	0.5	≥45	210	19	HMGCR, PPARA, ALDH2, SERPINA1, PPARG, VDR, ALB, JAK2, F2, CASP3, RBP4, AKT1, PCK1, FABP1, SCD, TNF, CYP2C19, IL6, GLUL

Table S3 GeneCard cut-off points

**GeneCards cutoff**	**Compound set fixed**	**MASLD-related genes retained**	**Shared targets (n)**	Overlapping targets retained
≥10	STP0.1 + PM300	315	35	PTPN2, IDO1, FABP1, SCD, TNF, GPBAR1, CYP2C19, IL6, GLUL, ALB, MAPK1, CES1, TTR, PPARG, EGFR, GSTA1, RORA, MET, PPARA, NR1H3, SOD2, RBP4, NR1H4, AKT1, NQO1, IGF1, HMGCR, CASP3, SERPINA1, VDR, PCK1, ALDH2, JAK2, ACADM, MMP9
≥30	STP0.1 + PM300	235	33	IDO1, FABP1, SCD, TNF, GPBAR1, CYP2C19, IL6, GLUL, ALB, MAPK1, CES1, TTR, PPARG, EGFR, RORA, MET, PPARA, F2, SOD2, RBP4, NR1H4, AKT1, NQO1, IGF1, HMGCR, CASP3, SERPINA1, VDR, PCK1, ALDH2, JAK2, ACADM, MMP9
≥45	STP0.1 + PM300	173	26	ALB, TTR, PPARG, MET, PPARA, F2, RBP4, NR1H4, AKT1, NR1H3, IGF1, HMGCR, CASP3, SERPINA1, VDR, PCK1, ALDH2, JAK2, ACADM, MMP9, FABP1, SCD, TNF, CYP2C19, IL6, GLUL
